# Study on the Wear Behavior Mechanism of SUS304 Stainless Steel During the Homogenization Process of LFP/NCM Slurry

**DOI:** 10.3390/ma18194457

**Published:** 2025-09-24

**Authors:** Xiangli Wen, Mingkun Bi, Lvzhou Li, Jianning Ding

**Affiliations:** 1 Institute of Technology for Carbon Neutralization, Yangzhou University, Yangzhou 225127, China; 18862295932@163.com (M.B.); dingjn@yzu.edu.cn (J.D.); 2 School of Mechanical Engineering, Yangzhou University, Yangzhou 225127, China

**Keywords:** SUS304 stainless steel, lithium iron phosphate, lithium nickel cobalt manganese oxide, wear behavior and regularity, wear evolution mechanism

## Abstract

During the homogenization process of lithium battery slurry, the slurry shearing process causes the surface of the homogenization equipment to wear and generate metal containing debris, which poses a risk of inducing battery self-discharge and even explosion. Therefore, inhibiting wear of homogenizing equipment is imperative, and systematic investigation into the wear behavior and underlying mechanisms of SUS304 stainless steel during homogenization is urgently required. In this study, lithium iron phosphate (LFP) and lithium nickel cobalt manganese oxide (NCM) cathode slurries were used as research objects. Changes in surface parameters, microstructure, and elemental composition of the wear region on SUS304 stainless steel under different working conditions were characterized. The results indicate that in the SUS304-lithium-ion battery slurry system, the potential wear mechanism of SUS304 gradually evolves with changes in load and rotational speed, following the order: adhesive wear (low speed, low load) → abrasive wear (medium speed, high load) → fatigue wear (high speed). Under high-load and high-rotational-speed conditions, oxidative corrosion wear on the ball–disc contact surface is particularly pronounced. Additionally, wear of SUS304 is more severe in the LFP slurry system compared to the NCM system. Macroscopic experiments also revealed that the speed effect is a core factor influencing the wear of SUS304, and the increase in its wear rate is more than twice that caused by the load effect. This study helps to clarify the wear behavior and wear mechanism evolution of homogenization equipment during the lithium battery homogenization process, providing data support and optimization direction for subsequent material screening and surface strengthening treatment of homogenization equipment components.

## 1. Introduction

Facing the challenges of global energy demand growth and low-carbon transition, lithium-ion batteries occupy a crucial position in the energy storage field due to high energy density [1], absence of memory effect [2], long cycle life [3], and relatively low self-discharge rate [4]. The performance of lithium batteries is affected by factors such as materials [5], preparation methods [6], and electrolytes [7,8]. Among these, the low energy density and coulombic efficiency of cathode materials are key parameters that restrict the overall performance of lithium-ion batteries [9,10,11]. Lithium iron phosphate (LFP) with high safety and long cycle life [12], and lithium nickel cobalt manganese oxide (NCM), with a high energy density [13], have been applied on a large scale in various fields. As a core link in battery manufacturing, the homogenization quality of battery slurry affects the uniform dispersion of active components, the compactness and structural uniformity of the electrode microstructure, and thus further influences the energy density and electrochemical performance of the battery [14,15]. Homogenizing equipment plays an important role in the process of dry, wet or semi-dry slurry preparation processes. The macroscopic process is characterized by the dispersion and uniform mixing of each component, which promotes the interaction between particles and forms a stable network structure from the microscopic point of view, and improves the performance of the battery [16,17].

As a new type of continuous mixing equipment, the twin-screw extruder can significantly shorten the mixing time compared with the traditional homogenizer [18], and can flexibly combine the screw elements according to the process requirements [19]. It is the key equipment for intelligent and high-efficiency homogenization in the future. However, due to long-term continuous stirring, the extruder components undergo varying degrees of wear. The generated metal wear debris not only contaminates the slurry but also induces battery self-discharge and even poses a risk of explosion [20,21]. Metal particles gradually dissolve and diffuse at the high-potential cathode, and dendrites precipitate at the low-potential anode, leading to diaphragm perforation and internal short circuits [22]. Additionally, Fe is oxidized to Fe_2_O_3_ and Fe_3_O_4_ [23]. Among these products, Fe^2+^ participates in the formation of an irregular solid electrolyte interphase (SEI) film [24]. while Fe^3+^ further exacerbates cation mixing, which causes electrode structure degradation and the generation of harmful impurities such as LiFeO_2_. This process accelerates interface degradation, resulting in an 8.6–16.3% capacity loss at high rates (5C) and a 3.5–12.8% capacity loss after 60 cycles [25,26]. Previous studies have shown that parameters such as screw structure and operating process jointly affect the degree of screw wear [27]. Simulation results indicate that the wear of twin screws is most severe at the screw flights [28], and as the rotational speed increases, the shear rate at the screw flights becomes higher, leading to more intense wear [29,30]. In the homogenizing process, the twin-screw is also affected by multiple factors to increase the shear stress, resulting in increased wear [31,32,33,34,35]. The local shear rate of the kneading screw is larger than that of the conveying screw, and the shear effect is stronger [31]. The difference in structural parameters such as pitch [32], helix angle and stagger angle [33] will also change the shear stress distribution. When the spiral gap is reduced by half, the shear stress is increased by nearly 2 times [34]. In addition, as the slurry filling degree inside the cavity increases from 25% to 100%, the compressive force between particles gradually increases, and the distribution of shear rate tends to be concentrated, resulting in the shear stress in the shear zone increasing by more than 2 times [35]. At present, the negative impact of wear in homogenizing equipment has attracted widespread attention, and extensive simulation work has been carried out on the optimization of component configurations and process parameters. However, the core factors that exacerbate wear during the twin-screw homogenization process remain unclear, and research on the macroscopic wear behavior regularity and the evolution process of wear mechanisms of core components in homogenizing equipment is still insufficient.

In this work, stainless steel (SUS304) was used as the friction pair material to simulate the wear behavior of SUS304 during the homogenization of LFP and NCM slurries through friction experiments under different loads and speeds. Through in-depth analysis of the surface parameters, microtopography, and elemental composition of the wear areas, it was found that as the load and rotational speed change in the SUS304-lithium battery slurry system, the potential wear mechanism of SUS304 exhibits an evolutionary process of “adhesive wear (low speed, low load) → abrasive wear (medium speed, high load) → fatigue wear (high speed)”. The speed effect is the core factor affecting the wear of SUS304, and the wear of SUS304 in LFP system is more severe than that in NCM slurry. This work fills the gap in the systematic investigations of macroscopic wear behavior in lithium-ion battery slurry homogenization equipment and also provides data support and optimization direction for the subsequent design and optimization of homogenization equipment.

## 2. Materials and Methods

### 2.1. Experimental Materials

The battery slurry is composed of active material LFP/NCM, polyvinylidene fluoride (PVDF), conductive carbon black (CB) and N-methyl pyrrolidone (NMP), all purchased from Suzhou Dodo Chemical Technology Co., Ltd. (Suzhou, China). The physicochemical parameters of LFP and NCM are shown in Table 1. Petroleum ether and absolute ethanol were purchased from Shanghai Aladdin Biochemical Technology Co., Ltd. (Shanghai, China), with purities surpassing 99%, in line with international standards [36]. The diameter of the SUS304 ball used in the experiment is 6 mm, the diameter of the SUS304 disc is 24 mm, and the thickness is 7.88 mm. The composition and physicochemical properties are shown in Table 2.

### 2.2. Experimental Methods

**Figure 1:**
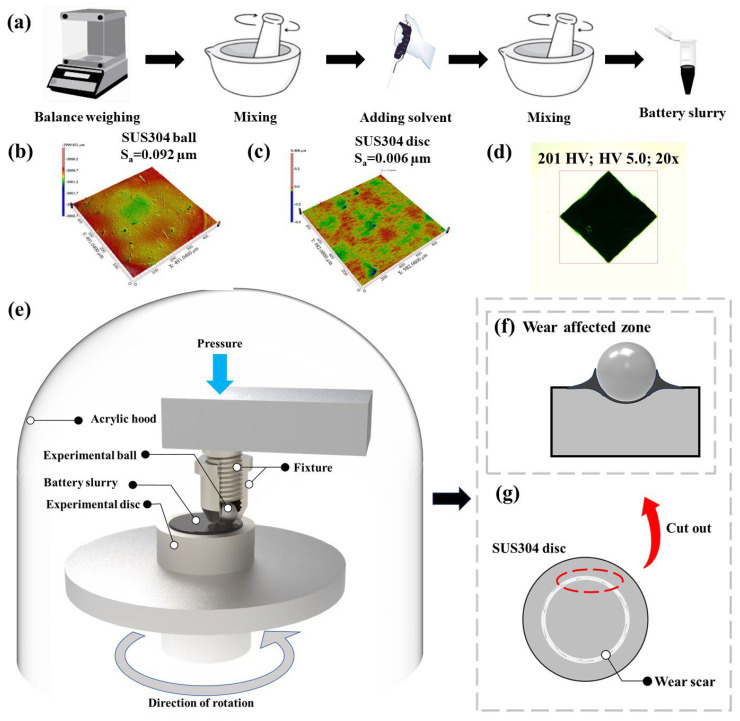
(**a**) Schematic diagram of the preparation process for lithium battery slurries LFP/NCM; surface S_a_ of SUS304 (**b**) balls and (**c**) discs before the experiment; (**d**) surface hardness of the disc; (**e**) schematic diagram of the point contact of the friction and wear tester device; (**f**) cross-sectional view of the ball–disc contact area; (**g**) schematic diagram of the disc wear area.

The battery slurry is composed of LFP/NCM:PVDF:CB = 8:1:1, and the preparation process is shown in Figure 1a. Before and after the experiment, the SUS304 balls and discs were sequentially ultrasonically cleaned in petroleum ether and absolute ethanol for 15 min each, then stored in absolute ethanol for later use. Figure 1b,c show that the initial surface roughness (S_a_) of the ball and disc is 0.092 µm and 0.006 µm, respectively, and the Vickers hardness of the disc is approximately 201 (Figure 1d). The friction experiments were conducted using an atmosphere friction and wear tester (WTM-2E, Lanzhou Zhongke Kaihua Technology Development Co., Ltd., Lanzhou, China), with schematics of its point-contact working mode and wear area shown in Figure 1e–g. All experiments were performed under the conditions of a rotating radius of 4 mm, room temperature of 25 °C, and relative humidity (RH) of 20%, with the experimental scheme detailed in Table 3. To ensure the reliability of results, each group of experiments was repeated at least three times. The surface morphology of the wear area was observed using an optical microscope (MSD380T, Maishidi Technology Co., Ltd., Dongguan, China) and a 3D white light interferometric profiler (SuperView W1, Shenzhen Zhongtu Instrument Co., Ltd., Shenzhen, China). The surface parameters of the wear areas were measured, including S_a_, wear scar depth, and wear volume (V), and the wear rates (*δ*) of the balls and discs were calculated separately [37]. The microstructure, element composition and content of the wear area were analyzed by field emission scanning electron microscopy (SEM) combined with energy dispersive spectrometer (EDS) (GeminiSEM 300, Carl Zeiss, Oberkochen, Germany).

## 3. Results and Discussion

### 3.1. LFP/NCM Slurry Analysis

The battery slurry in this research is a multiphase suspension, where the dispersed phase (consisting of LFP/NCM and CB) is uniformly dispersed in the PVDF-NMP colloid (continuous phase) [38,39]. During macroscopic flow, the relative movement between particles in the dispersed phase and the continuous phase gives rise to notable interfacial velocity gradients and shear actions. In essence, the particles are affected by the combined effects of sedimentation, Brownian motion, and shear-induced directional alignment, and such behavior is governed by the flow conditions of the battery slurry [40,41]. Viscosity, a key parameter in lithium-ion battery slurry preparation, quantifies the flow resistance of fluids. It exerts a direct impact on the slurry’s dispersion stability, rheological characteristics, process compatibility, and the ultimate performance of the electrode [42]. Its defining formula is presented below:

(1) $$\eta =\frac{\tau }{\dot{\gamma }}$$

(2) $$\tau =\frac{F}{S}$$

(3) $$\dot{\gamma }=\frac{\mathrm{du}}{\mathrm{dy}}$$

where *η* is the viscosity (Pa·s), *τ* is the shear stress (Pa), $\dot{\gamma }$ is the shear rate (s^−1^), *F* is the shear force (N), *S* is the contact area (m^2^), *du* is the velocity difference between adjacent fluid layers perpendicular to the flow direction (m/s), and *dy* is the distance between the two layers (m).

Studies have shown that the rheological properties of the slurry are determined by the interaction between the active material and the binding material [43]. The LFP active material has a typical three-dimensional network olivine crystal structure [44], which tends to agglomerate (Figure S1a), resulting in local concentration inhomogeneity and the formation of “particle-enriched regions”. During the experiment, large-sized LFP agglomerates may aggravate the wear on the surface of the friction pair. In contrast, the NCM active material exhibits a spherical or quasi-spherical secondary agglomerate morphology, with clear interfaces and distinct boundaries between particles, showing a more regular spherical shape (Figure S1b). This morphology promotes the uniform distribution of particles and enables more uniform movement of the two phases.

As shown in the slurry rheological curves in Figure S2, within the shear rate range of 0.1–3000 s^−1^, both slurries exhibit shear-thinning behavior, i.e., the viscosity decreases monotonically with increasing shear rate. At low shear rates, the slurry shows high viscosity due to strong interparticle forces (e.g., van der Waals forces or the adsorption of binders). The slurries show high viscosity characteristics and reduced fluidity. This causes small-scale aggregation of particles inside the slurries; these aggregated particles are pushed away under the shear force between friction pairs, leading to local dry friction contact. At high shear rates, however, the strong shear force disrupts the network structure formed between materials, causing the particles to rearrange into a more ordered structure parallel to the shear field [45,46,47]. This rearrangement reduces the internal frictional resistance of the slurry and decreases its load-bearing capacity, thereby increasing the probability of direct contact between the rough peaks in the contact area of the friction pair and exacerbating wear.

Meanwhile, Figure S2 also indicates that under the same shear rate condition, the viscosity of the NCM slurry is significantly higher than that of the LFP slurry. This implies that the NCM system has greater fluid flow resistance, and its coefficient of friction (COF) may be higher than that of the LFP system.

### 3.2. Tribological Properties of LFP/NCM Slurry Under Different Working Conditions

**Figure 2:**
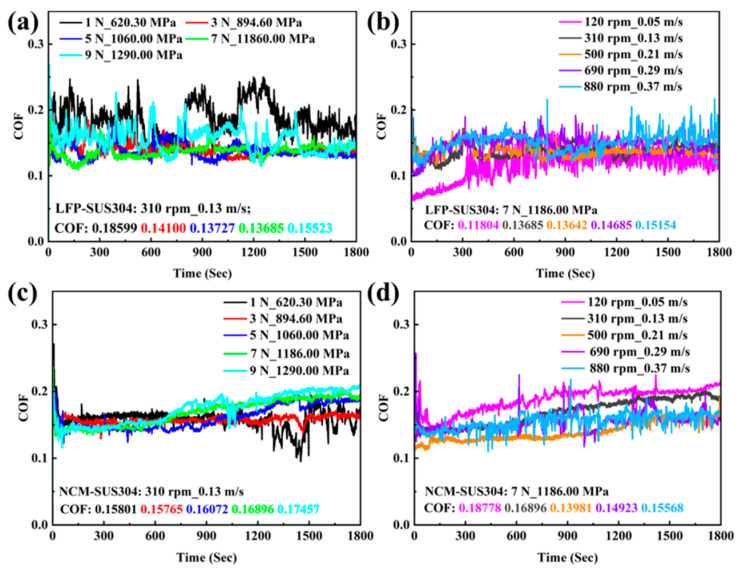
(**a**,**b**) LFP and (**c**,**d**) NCM slurry system, SUS304 in (**a**,**c**) load 1–9 N and (**b**,**d**) speed 120–880 rpm COF over time. All experiments were performed at 50% RH, 25 °C, and a rotation radius of 4 mm.

Figure 2 shows the curves of COF versus time for SUS304 under different working conditions during the homogenization process of LFP and NCM slurries. The results indicate that the COF curves of all samples exhibit obvious fluctuations, which may be attributed to the random “protrusions” generated when slurry particles or wear debris enter the ball–disc contact area during homogenization [36]. Figure 2a,b indicates that the COF of the LFP slurry fluctuates more significantly. Under low load (1 N), after repeated running-in between the ball and the disc, the COF fluctuates drastically, with its instantaneous value reaching a maximum of 0.250. As the load increases from 3 N to 7 N, the COF remains at ~0.140 and is relatively stable. When the load is further increased to 9 N, obvious material removal and plastic deformation occurred in SUS304 during homogenization, and COF increased to 0.155. In addition, at a rotational speed of 120 rpm, SUS304 undergoes stable running-in in the first 300 s. Then, due to the high viscosity of the slurry at low speed, part of the slurry is pushed away from the wear area by the ball, resulting in local “dry friction” in the ball–disc contact area, which causes continuous and severe fluctuations in COF with an average COF of 0.118. As the rotational speed continues to increase, within the range of 310 to 690 rpm, the COF rises steadily from 0.135 to 0.146 and remains relatively stable. When the rotational speed increases to 880 rpm, part of the slurry is thrown out of the ball–disc contact area under the action of centrifugal force, which enhances the impact of abrasive particles on the wear region; as a result, the average COF increases to 0.152 with more drastic fluctuations.

Figure 2c,d shows the COF curves of the NCM slurry under different working conditions, where the COF is more stable compared to the LFP slurry. At a load of 1 N, the contact pressure is low, and the COF fluctuates significantly, with a variation trend similar to that of the LFP slurry. As the load gradually increases to 9 N, the average COF also increases to 0.175. The COF of the SUS304-NCM system is relatively low and stable in the first 600 s. As the homogenization process continues, the COF gradually increases, which may be due to the increase in slurry solid content caused by wear debris generated during the running-in process, enhancing the direct contact between debris/slurry particles and the friction pairs. With the increase in rotational speed, the average COF decreases first and then increases, with the most stable and relatively low COF at 500 rpm. The results indicate that the COF of the SUS304-LFP/NCM system is relatively stable with smaller fluctuations at 3–7 N and 310–500 rpm. The COF of the NCM system is higher than that of the LFP system, which may be attributed to the differences in the active materials of the two slurries. The size and shape of particles inside the slurry can adjust the yield stress, viscoelasticity, and shear-thinning behavior of the slurry by altering the particle-binder interface interaction, thereby leading to differences in the tribological properties of the slurry [48,49].

**Figure 3:**
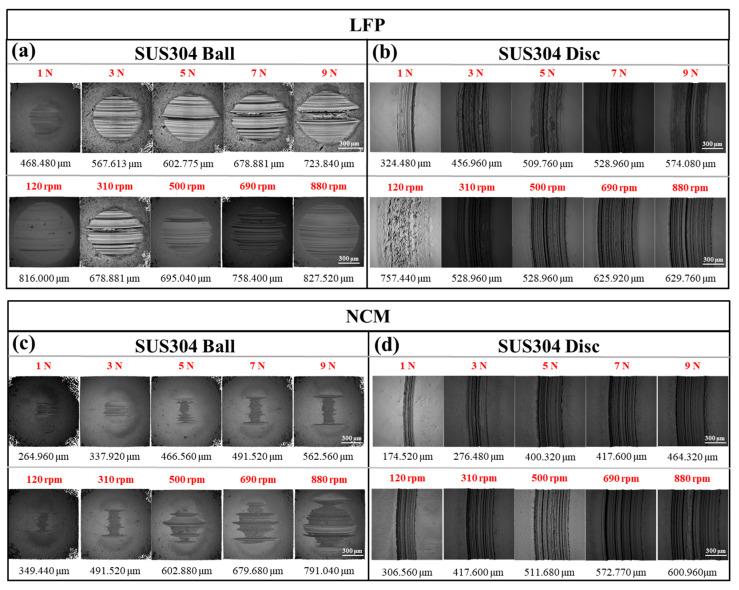
(**a**,**b**) LFP and (**c**,**d**) NCM slurry system after friction experiment, SUS304 (**a**,**c**) ball and (**b**,**d**) disc under different load (1–9 N) and speed (120–880 rpm) light microscope.

To gain a deeper understanding of the wear behavior in the SUS304-LFP/NCM system, the ball and disc wear areas were observed and measured by an optical microscope (Figure 3). As shown in Figure 3a,b, in the LFP slurry system, the wear scar diameter (WSD) of the SUS304 balls and the wear scar width (WSW) of the discs tend to increase with increasing load. At 9 N, the WSD and WSW increase by 54.51% and 76.92%, respectively, compared to those at 1 N. The load effect influences the ball–disc contact stress. As the load increases, plastic deformation occurs on the ball surface, and uneven distribution of contact stress leads to insufficient running-in in local areas, causing the wear scar on the ball surface to gradually evolve from a circular to an elliptical shape. At 120 rpm, due to the excessively high viscosity of the slurry, local “dry friction” forms between the ball and the disc, resulting in significant material removal from the ball surface. The WSD reached 816 µm, and numerous peeling pits appear on the disc surface with a WSW of 757.44 µm. As the rotation speed increased to 310 rpm, the shear-thinning property of the slurry enhanced the fluidity of the slurry and made it evenly distributed in the ball–disc contact area. WSD and WSW reached their minimum values, which were 16.80% and 30.16% lower than those at 120 rpm. As the rotation speed further increased to 880 rpm, part of the slurry was thrown out and its wear increased significantly. Compared with 310 rpm, WSD and WSW increased by 21.89% and 19.06%, respectively.

The results show that the wear scar size of the NCM slurry system is much smaller than that of the LFP system, and the wear scar on the ball presents an irregular strip shape with extensive material removal on both sides of the edge (Figure 3c,d). The fundamental reason for the differences in the wear properties of the slurries lies in the fact that, in the contact area, agglomerated LFP particles come into direct contact with the friction pairs, generating severe cutting action, whereas spherical NCM particles play a rolling role in the contact area [50]. With the increase in load, the variation trend of NCM slurry was similar to that of the LFP slurry. WSD and WSW increased from 264.96 µm and 174.52 µm at 1 N to 562.56 µm and 464.32 µm at 9 N, respectively. Different from the LFP system, the wear scar of the ball is irregularly banded at 120 rpm, and there are no obvious peeling pits on the disc surface. The WSD and WSW are only 264.96 µm and 306.56 µm, which are 57.18% and 59.53% lower than those in the LFP system, respectively. With increasing rotational speed, the wear scar dimensions on both the ball and disc exhibited a progressive gradient increase. At 880 rpm, WSD and WSW increased by 126.37% and 125.57%, respectively, compared with 120 rpm, and the wear scar of the ball gradually transitioned to a regular circular shape with the increase in rotational speed. The tribological properties of the two slurry systems were compared, and it was found that the COF of LFP slurry is lower, but the wear of SUS304 is more severe. This is mainly because under the same conditions, the actual ball–disc contact area in the LFP system was larger, and the corresponding contact pressure was much lower than that in the NCM system, which in turn resulted in a lower COF in the LFP system [51].

**Figure 4:**
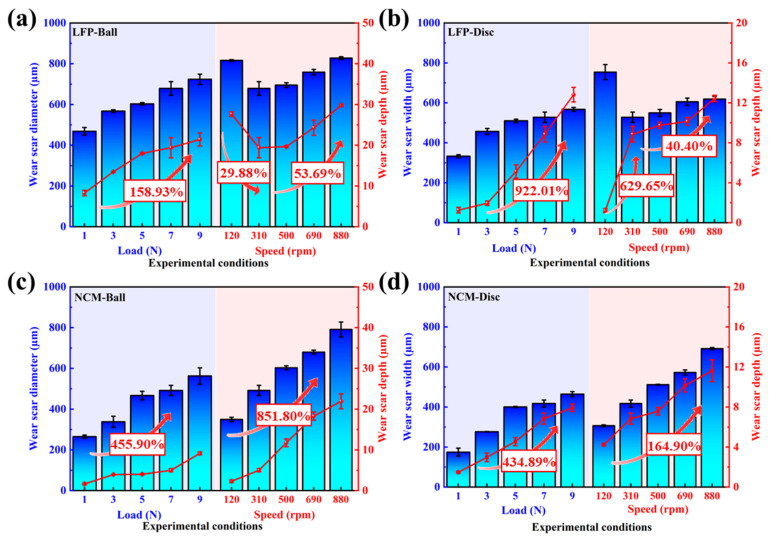
After the friction experiment of (**a**,**b**) LFP and (**c**,**d**) NCM slurry system, the WSD and wear depth comparison of (**a**,**c**) SUS304 ball, the WSW and wear depth comparison of (**b**,**d**) disc.

**Figure 5:**
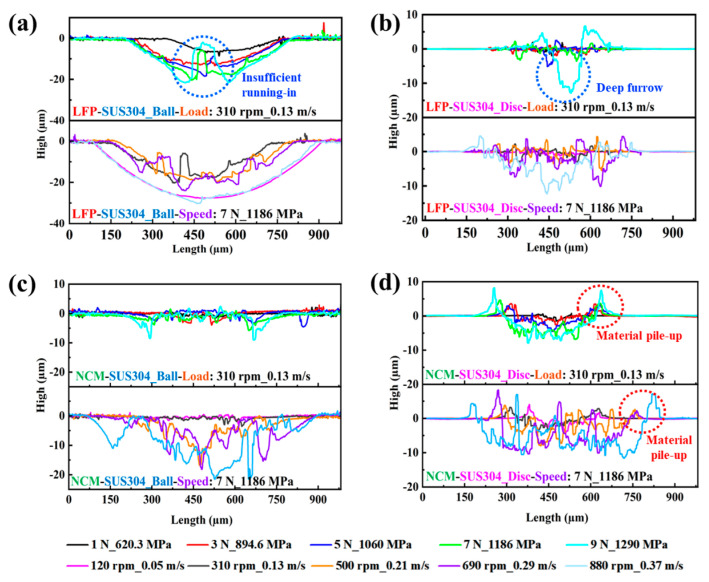
(**a**,**b**) LFP and (**c**,**d**) NCM slurry system friction experiment, under different experimental conditions (**a**,**c**) SUS304 ball and (**b**,**d**) plate grinding depth curve. All experiments were performed at 50% RH, 25 °C, and a rotation radius of 4 mm.

To analyze the changes in surface parameters of the wear regions after friction tests and further reveal the influence law of working conditions on the wear characteristics of SUS304 under different slurry systems, the surface topography parameters of the balls and discs were measured using a 3D white light interferometric profiler. The results are shown in Figure 4 and Figure 5 and Table 4. The results indicate that under the same slurry system, the wear scar depth and V of the wear scars on the ball and the disc both show an increasing trend with the increase in load and rotational speed. In the LFP system, the wear scar depth of the ball increased from 8.267 µm under low load (1 N) to 21.405 µm under high load (9 N), an increase of nearly 158.93% (Figure 4a). However, the wear scar depth of the disc was only 1–2 µm under low loads (1–3 N), and doubles to 5.122 µm at 5 N. The reason for this is that at medium to high loads (5–9 N), plastic deformation occurs on the ball surface, precluding adequate running-in. This elevation in contact pressure on the disc results in the formation of deeper plowing grooves (Figure 5a,b). As the load rises to 9 N, the plowing grooves on the disc surface become even deeper, and the wear depth exceeds 9 times the value observed at 1 N (Figure 4b).

Under rotational speed conditions, due to the severe wear of the ball at 120 rpm, its wear scar depth reaches 27.65 µm, while the disc surface is covered with numerous material peeling pits without obvious furrows, resulting in slight wear with a wear scar depth of only 1.21 µm. When the rotational speed increased to 310 rpm, the wear scar depth of the ball decreased to 19.39 µm with the decrease in WSD, but the wear scar depth of the disc increased by nearly 6 times, up to 8.85 µm. When the rotational speed further increased to 880 rpm, the wear scar depths of the ball and disc increased by 53.69% and 40.40%, respectively, compared to those at 310 rpm. In addition, in the NCM system, the wear scar depth of the ball at 1 N was 1.644 µm. As the load increased to 9 N, wear on the ball intensifies only at the edge of the wear scar, with no significant change in the wear scar depth in the central area (Figure 5c), and the wear scar depth increases by 455.90% compared to that at 1 N (Figure 4c). The wear scar depth of the disc at 1 N is 1.48 µm, and when the load increases to 9 N, the wear scar depth increases to 7.927 µm, nearly a 4-fold increase (Figure 4d), with corresponding material accumulation observed on both sides of the worn area (Figure 5d). This is because the load effect affects the ball–disc contact stress, causing plastic deformation on the SUS304 surface, and the displacement of surface materials and wear-generated debris accumulate on both sides of the wear scar [52]. When the rotational speed reaches 500 rpm, the wear scar depth of the ball increases by ~9.5 µm compared to that at 120 rpm. It is observed that obvious wear occurs in the central area, and the wear scar evolves from band to circle, which indicates that the speed effect is the core factor affecting the wear of SUS304. With a further increase in rotational speed, at 880 rpm, the wear scar depths of the ball and disc increased by 851.80% and 164.90%, respectively, compared to those at 120 rpm.

To more accurately compare the wear conditions of SUS304 balls and discs under different test conditions, the *δ* of SUS304 balls and discs after friction experiments was further calculated based on the surface parameters of the wear areas. The calculation formula is as follows [37]:

(4) $$\delta_{ball}=\frac{V}{F_{N}\cdot \nu \cdot t}=\frac{V}{{2\cdot \pi \cdot R\cdot \nu_{rpm}\cdot F}_{N}\cdot t}$$

(5) $$\delta_{disc}=\frac{216\cdot V}{1750000\cdot t\cdot \nu_{rpm}\cdot F_{N}}$$

where *F_N_* is the contact pressure (N), ν is the linear velocity (m/s), *t* is the experimental duration (s), *R* is the rotating radius (m), and $\nu_{rpm}$ is the experimental rotational speed (rpm).

**Figure 6:**
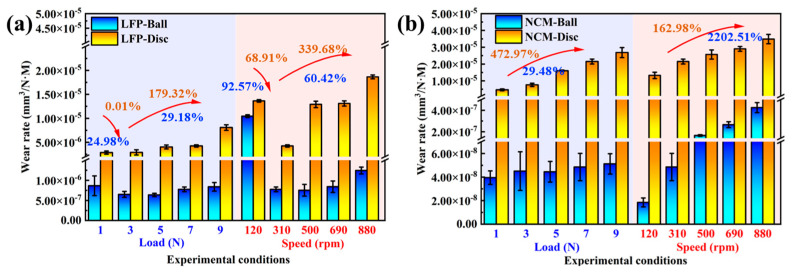
Comparison of δ for SUS304 spheres and discs under different experimental conditions in (**a**) LFP and (**b**) NCM slurry systems.

The calculated results of *δ* are shown in Figure 6. From Figure 6a and Figure S3, it can be seen that in the LFP system, *δ* shows a trend of first decreasing and then increasing with the increase in load and rotational speed. Under low load (1 N) conditions, the ball and disc undergo repeated running-in, with the *δ* of the ball and disc being 8.662 × 10^−7^ mm^3^·N^−1^·m^−1^and 2.853 × 10^−6^ mm^3^·N^−1^·m^−1^, respectively. When the load increases to 3 N, although the wear volume of the ball and disc increases compared to that at 1 N, the stress distribution tends to be uniform due to increased contact pressure, resulting in more stable and uniform wear. The *δ* of the ball and disc decreases by 24.98% and 92.57%, respectively, reaching the minimum values. When the load increases to 9 N, the load-bearing capacity of the slurry approaches its limit, leading to intensified wear of the ball and disc, and the *δ* of the disc increases nearly 3-fold compared to that at 1 N. In the LFP system at low rotational speed (120 rpm), severe wear occurs on the ball and disc, with their *δ* being 1.045 × 10^−5^ mm^3^·N^−1^·m^−1^ and 1.364 × 10^−5^ mm^3^·N^−1^·m^−1^, respectively. Similarly, when the rotational speed increases to 310 rpm, the wear scars of the ball and disc are the smallest, with *δ* being 7.759 × 10^−7^ mm^3^·N^−1^·m^−1^ and 4.241 × 10^−6^ mm^3^·N^−1^·m^−1^, respectively. As the rotation speed further increased to 880 rpm, the *δ* of the ball decreased by 92.57% compared with 120 rpm. However, due to the reduced load-bearing capacity of the slurry, the wear of the disc intensifies, with its *δ* increasing by 36.73%.

As shown in Figure 6b and Figure S4, in the NCM slurry system, the *δ* of the balls and discs increase continuously with the increase in load and rotational speed. Under the condition of 9 N, the *δ* values of the balls and discs are 22.48% and 472.92% higher than those at 1 N, respectively; at 880 rpm, the *δ* value of the balls is nearly 22 times higher than that at 120 rpm. The speed effect significantly accelerates the relative motion and interaction frequency of the abrasive particles and the contact area [53], making the *δ* of SUS304 change more significantly with the increase in speed, which proves that the speed effect is the core factor affecting the wear of SUS304. This result is consistent with the simulation results showing that high rotational speeds exacerbate screw wear.

Comparative analysis reveals that compared with the NCM system, the *δ* of balls in the LFP system is higher, while the *δ* of discs in the LFP system is lower. This may be because material removal from the ball surface is more obvious in the LFP system, with the wear scar being circular or elliptical (Figure 3a). The larger ball–disc contact area reduces the actual contact pressure, resulting in the *δ* of the SUS304 disc in the LFP system being much smaller than that in the NCM system. The results indicate that in the LFP slurry system under 3 N-310 rpm conditions, there is no significant running-in phenomenon like that under 1 N (310 rpm) or 120 rpm (7N); the COF is relatively low and stable, and the wear of the ball and disc is minimal. Therefore, this condition is more suitable for the homogenization process of LFP slurry. Compared with the NCM slurry system, although the COF is as high as 0.188 at 120 rpm, the ball and disc are stable and the wear of the ball and disc is the smallest. The NCM slurry is more suitable for homogenization at 1 N (310 rpm) and 120 rpm (7 N). The results provide data support for the selection of the optimal working condition parameters of the homogenization equipment in the process of large-scale lithium slurry homogenization.

### 3.3. Micromorphology and Composition Analysis of Wear Area

**Figure 7:**
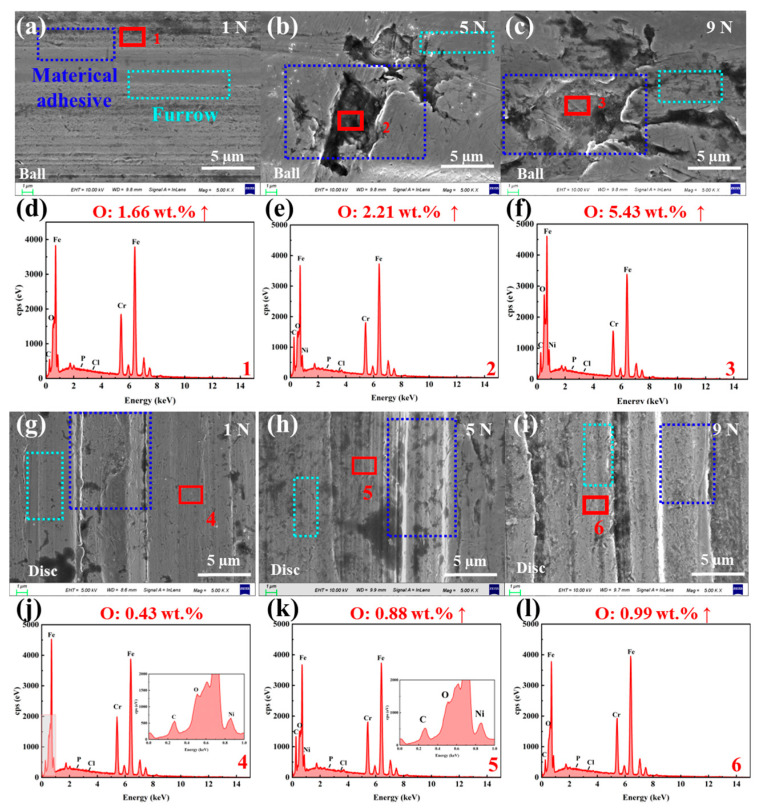
SEM micro morphologies of the wear areas on SUS304 (**a**–**c**) balls and (**g**–**i**) discs in the SUS304-LFP slurry system under different loads (1, 5, and 9 N), and EDS elemental composition analyses of the wear areas on (**d**–**f**) balls and (**j**–**l**) discs.

To further reveal the wear mechanisms of SUS304 under different slurry systems, SEM-EDS was used to analyze the micromorphology and compare the surface compositions of the wear areas on the balls and discs before and after the experiments. The initial morphology and composition of the ball and disc are shown in Figure S5, and the surface O element content is ~0.4 wt.%. Figure 7 presents the micro morphology of the wear regions and the variation in O element content for the SUS304-LFP slurry system under different loads, while all elements detected in the wear regions are shown in Figure S6 and Table S1. It can be seen from Figure 7a–c that the LFP slurry system has significant material removal on the ball surface at 1 N, a small amount of shallow furrows are distributed, and the surface of the wear area is smooth. When the load increases to 5 N, obvious material detachment pits appear on the ball surface; when the load further increases to 9 N, the wear is significantly aggravated, with a large area of pits and furrows forming on the surface. With the increase in load, the oxygen (O) element content in the wear regions gradually increases from 0.4 wt.% to 5.43 wt.% (Figure 7d–f), which further confirms that oxides are generated in the wear regions and their content continues to rise. From Figure 7g–i, it can be observed that as the load increases, abrasive particles generated on the disc surface and slurry particles enter the worn area, resulting in obvious material peeling and numerous furrows. Under high load, “three-body abrasive wear” is the dominant mechanism [54]. Similarly, as the load increases, the O element content in the wear area of the disc surface increases from 0.4 wt. % to about 1 wt. %, which is consistent with the change rule of the element content on the ball surface (Figure 7j–l).

**Figure 8:**
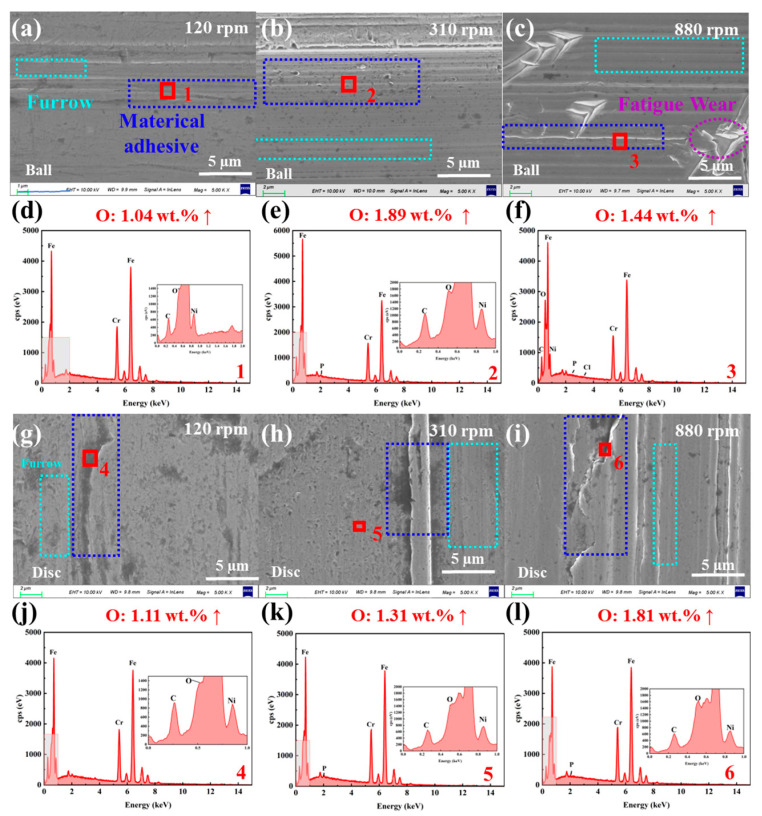
SEM micro morphologies of the wear areas on (**a**–**c**) balls and (**g**–**i**) discs in the SUS304-LFP slurry system under different rotational speeds (120, 310, and 880 rpm), and EDS elemental composition analyses of the wear areas on (**d**–**f**) balls and (**j**–**l**) discs.

Figure 8a–c show that the surface of the LFP slurry system is smooth at low speed (120 rpm), with only a small number of pits and furrows. Figure 8d,e shows that with the increase in rotational speed, the ball surface wear becomes more severe, accompanied by the emergence of numerous grooves, and the surface oxygen content rises steadily to 1.89 wt.% (details of elements detected in the wear areas are provided in Figure S6 and Table S1). At high rotational speed (880 rpm), triangular peeling areas form on the ball surface, which may consist of continuously propagating cracks, indicating that fatigue wear may occur under high-speed conditions. However, the O content in this area only increases slightly (Figure 8f). Figure 8g–i reveal that as the rotational speed increases, material removal from the SUS304 disc surface intensifies, and the surface furrows deepen continuously. The O content in the worn area increases from 0.4 wt.% to 1.81 wt.%, indicating that oxidative corrosion wear also occurs to a certain extent (Figure 8j–l). At high speed (880 rpm) and high load (9 N), the ball and disk surfaces exhibit more serious oxidative corrosion wear. Thus, in the LFP system, the wear mechanism of SUS304 undergoes a gradual transition: it exhibits characteristics of adhesive wear under low-speed and low-load conditions, evolves into abrasive wear under medium-speed and high-load conditions, and even shows a tendency to shift toward fatigue wear under high-speed conditions.

**Figure 9:**
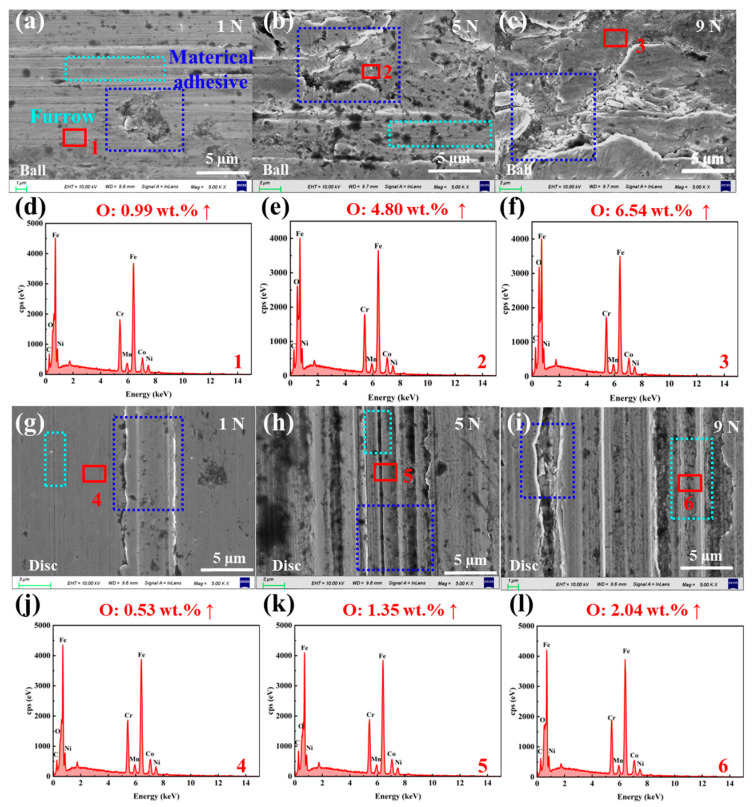
SEM micro morphologies of the wear areas on SUS304 (**a**–**c**) balls and (**g**–**i**) discs in the SUS304-NCM slurry system under different loads (1, 5, and 9 N), and EDS elemental composition analyses of the wear areas on (**d**–**f**) balls and (**j**–**l**) discs.

**Figure 10:**
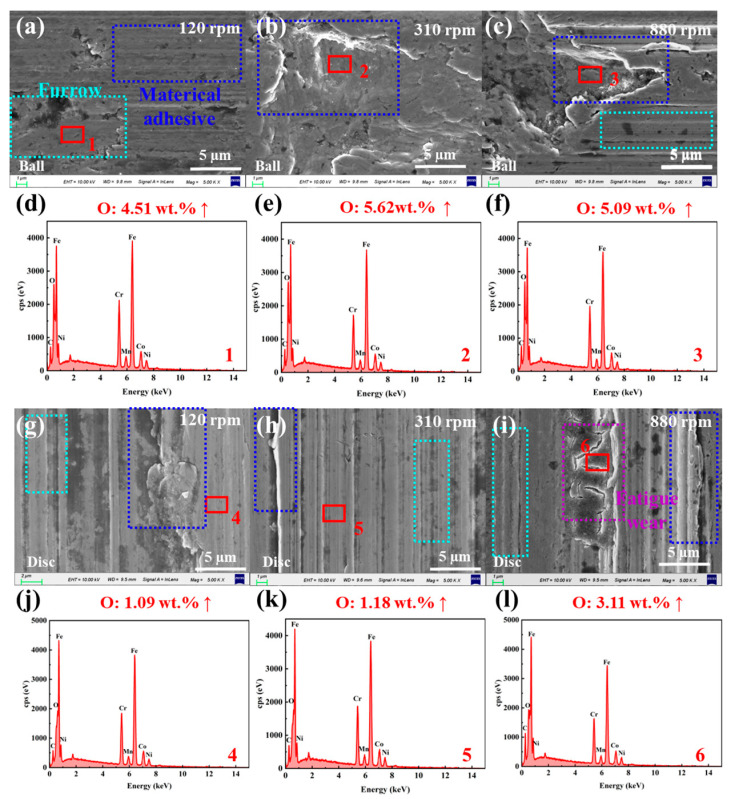
SEM micro morphologies of the wear areas on (**a**–**c**) balls and (**g**–**i**) discs in the SUS304-NCM slurry system under different rotational speeds (120, 310, and 880 rpm), and EDS elemental composition analyses of the wear areas on (**d**–**f**) balls and (**j**–**l**) discs.

Figure 9 and Figure 10 show the microstructure of the wear regions and the O element composition analysis of the SUS304 balls and discs during the homogenization process of the NCM slurry. All elements detected in the wear regions are supplemented in Figure S7 and Table S2. As observed in Figure 9a–c, the worn areas on the balls consist of material peeling pits and a small amount of furrows. With increasing load, material peeling becomes more severe, and the O element content gradually increases from 0.4 wt. % to 6.54 wt. % (Figure 9d–f). Figure 9g,h shows that there is only slight material removal on the surface of the disc under low load, and the wear is relatively small. As the load gradually increases to 9 N, the disc surface exhibits significant characteristics of “three-body abrasive wear”, with an increased number of furrows and further intensified plastic deformation. The content of O element in the wear area is shown in Figure 9j–l. As the load increases from 0.4 wt. % to about 2 wt. %, a certain degree of oxidative corrosion occurs.

Figure 10a–c reveal that in the NCM system at low rotational speed, a small amount of furrows and pits left by material peeling appear on the ball surface, with the O element content in the pits reaching 4.51 wt.% (Figure 10d). With the increase in rotational speed, the peeling pit gradually evolved into a large-scale, deeper “pocket” shaped pit, the material removal was intensified and accompanied by a large number of deeper furrows, and the content of O element gradually increased to 5.09 wt. % (Figure 10e,f). The micro-morphology of the wear area of the disc changes with the rotation speed as shown in Figure 10g–i: The micromorphology of the disc’s worn area changes with rotational speed as shown in Figure 10g–i: material wear and spalling (120 rpm) → emergence of numerous deep furrows (310 rpm) → occurrence of fatigue cracks (880 rpm). The O content in the wear area increases from 0.4 wt.% to about 3 wt.% (Figure 10j–l). Similarly, in the NCM slurry system, the wear behavior exhibits characteristics of adhesive wear under low-speed and low-load conditions, followed by a gradual transition process that progresses sequentially to abrasive wear and then to fatigue wear. It was found that the content of O element on the surface of the ball was higher than that on the surface of the disc under the two slurry systems, which is because the continuous wear of the balls causes the SUS304 substrate to continuously react with active substances in the slurry, generating a large amount of oxides. Under the conditions of high speed (880 rpm) and high load (9 N), the oxidative corrosion wear on the ball and disk surfaces is more severe.

### 3.4. Analysis of Wear Mechanism

**Figure 11:**
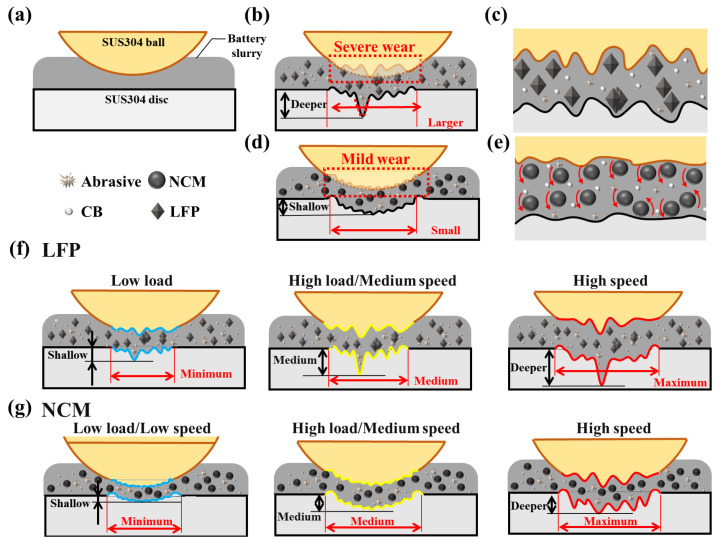
Wear mechanism of SUS304-LFP / NCM. (**a**) Schematic diagram of contact form before friction experiment, (**b**,**d**) macro wear diagram and (**c**,**e**) micro mechanism diagram of ball and disc wear area under (**b**,**c**) LFP and (**d**,**e**) NCM slurry system, the wear mechanism of (**f**) LFP and (**g**) NCM slurry system under different working conditions.

The wear mechanisms of the SUS304-LFP/NCM slurry system under different working conditions are illustrated in Figure 11. The surface of the initial SUS304 ball and disc is relatively smooth (Figure 1a). During the experiment, “three-body abrasive wear” occurs in the worn area under the combined action of slurry particles and wear debris generated during the experiment. When the LFP slurry is used as the medium (Figure 11b,c), due to the olivine-like crystal structure of the LFP particles, the agglomerated particles are in direct contact with the friction pair during the sliding process, which plays a significant cutting role, causing more severe wear on the surface of the ball and disc than the NCM slurry. The wear spot of the ball is round or oval, and the wear area is mainly surface contact, S_a_ is larger.

In the NCM slurry system (Figure 11d,e), due to the rolling effect of NCM particles in the contact area, the friction mode is transformed from sliding to rolling [50,55]. This results in only slight wear on the balls, forming strip-shaped wear scars. The ball and disc form an approximately line contact in the worn area, and the ball–disc contact pressure is more concentrated, leading to sufficient wear in the disc contact area with a smaller S_a_. Figure 11f shows that in the LFP slurry system, as the load and rotational speed increase, material removal from the ball surface intensifies, and the ball–disc contact area continues to expand. Because the surface of the ball is not fully run-in, the contact pressure between the ball and the disc is unevenly distributed, and deep furrows are generated and deepened in the local area of the disc. In the NCM slurry system (Figure 11g), with the continuous deterioration of the working conditions, the wear of the ball and disc gradually increased, and the wear spot of the ball transitioned to a circular shape. A comparison of the wear conditions of the two slurries indicates that SUS304 undergoes more severe wear in the LFP system. However, under both slurry systems, the wear mechanism of SUS304 exhibits a gradual transition process: it may manifest as adhesive wear in the initial stage, followed by a sequential development into abrasive wear and then fatigue wear. Meanwhile, a large number of oxides are formed on the surface of the wear area of the ball and disk.

## 4. Conclusions

By simulating the homogenization process of LFP and NCM slurries, this study systematically investigates the wear patterns of SUS304 stainless steel under different operating conditions and reveals the evolutionary process of its wear mechanisms. The key research findings are as follows:

(1) The potential wear mechanisms of the SUS304-lithium-ion battery slurry system exhibit a progressive evolution: adhesive wear (low rotational speed, low load) → abrasive wear (medium rotational speed, high load) → fatigue wear (high rotational speed). Additionally, under high-load and high-rotational-speed conditions, oxidative corrosion wear on the ball–disc contact surface is particularly pronounced.

(2) In the SUS304-lithium-ion battery slurry system, the effect of rotational speed on accelerating the wear rate of SUS304 is far more significant than that of load. As the rotational speed increases, the shear-thinning property of the slurry becomes more prominent, enhancing its fluidity. This increases the interaction frequency between particles and wear debris within the slurry and the surface of the SUS304 material, thereby amplifying the effect of “three-body abrasive wear”. Meanwhile, the crystal structure characteristics of the cathode material (LFP/NCM) are closely related to the wear behavior of SUS304.

(3) The SUS304-LFP slurry system is suitable for homogenization under the condition of 3 N (load)-310 rpm (rotational speed), while the SUS304-NCM slurry system is more suitable for homogenization under either 1 N-310 rpm or 7 N-120 rpm.

This study focuses on the equipment wear issue during the homogenization of two lithium-ion battery slurries (LFP and NCM). It systematically explores the wear behavior patterns and wear mechanism evolution of homogenization equipment under different operating conditions, optimization directions for subsequent material selection and surface strengthening treatment of key equipment components.

## Figures and Tables

**Table 1:** Physicochemical parameters of LFP and NCM.

Project	Parameters	
	LFP	NCM
**Particle size (µm)**	12.46	11.21
**Tapped density (g/cm^3^)**	1.64	2.32
**pH**	9.33	11.58
**Specific surface area (m^2^/g)**	7.54	0.28

**Table 2:** Composition and Physicochemical Parameters of SUS304.

Components (wt.%)		Materialized	Parameters
**Fe**	67–71.5	**σ_b_ (MPa)**	≥515–1035
**Cr**	17.5–19.5	**σ_0.2_ (MPa)**	≥205
**Ni**	8–10.5	**HV**	201
**C**	≤0.07	**ρ** ** (20 °C, g/cm^3^)**	7.93
**Mo**	----	**Melting point (** **℃** **)**	1398~1454
**Ti**	----	**ν**	0.3
**Others**	≤2.29	**Modulus of elasticity (20 °C, KN/mm^2^)**	193

**Table 3:** Parameters related to friction test.

Project	Parameters	
**Slurry**	LFP/NCM	
**Constant parameter**	310 rpm (0.13 m/s)	7 N (1186 MPa)
**Control parameter**	1 N (620.3 MPa), 3 N (894.6 MPa), 5 N (1160 MPa), 7 N (1186 MPa), 9 N (1290 MPa)	120 rpm (0.05 m/s), 310 rpm (0.13 m/s), 500 rpm (0.21 m/s), 690 rpm (0.29 m/s), 880 rpm (0.37 m/s)
**Temperature**	25 °C	
**Humidity**	20% RH	
**Rotating radius**	4 mm	
**Sample addition volume**	200 μL	
**Friction pair**	SUS304 ball–disc point contact	

**Table 4:** WSD, WSW, S_a_, wear scar depth and V of SUS304 ball and disc wear area after friction test in LFP and NCM slurry system.

Slurry	Working Conditions			SUS304 Ball				SUS304 Disc			
				WSD (µm)	S_a_ (µm)	Depth (µm)	V (µm^3^)	WSW (µm)	S_a_ (µm)	Depth (µm)	V (µm^3^)
**LFP**	**Load**	**310 rpm**	**1 N**	468.480	1.781	8.267	2.027 × 10^5^	324.480	0.1790	1.254	1.290 × 10^4^
			**3 N**	567.613	3.246	13.473	4.562 × 10^5^	456.960	0.3560	1.950	3.919 × 10^4^
			**5 N**	602.775	3.899	17.995	7.464 × 10^5^	509.760	0.7124	5.122	9.077 × 10^4^
			**7 N**	678.881	4.683	19.390	1.271 × 10^6^	528.960	1.0926	8.858	1.342 × 10^5^
			**9 N**	723.840	5.397	21.405	1.768 × 10^6^	574.080	3.4209	12.816	3.284 × 10^5^
	**Speed**	**7 N**	**120 rpm**	816.000	6.747	27.653	6.582 × 10^6^	757.440	0.2270	1.214	1.671 × 10^5^
			**310 rpm**	678.881	4.683	19.390	1.271 × 10^6^	528.960	1.0926	8.858	1.342 × 10^5^
			**500 rpm**	695.040	5.256	19.660	1.991 × 10^6^	558.720	2.7162	9.745	6.588 × 10^5^
			**690 rpm**	758.400	5.571	24.299	3.076 × 10^6^	625.920	2.3644	10.140	9.228 × 10^5^
			**880 rpm**	827.520	7.031	29.801	5.803 × 10^6^	629.760	2.9017	12.437	1.675 × 10^6^
**NCM**	**Load**	**310 rpm**	**1 N**	264.960	0.273	1.644	9.259 × 10^3^	192.000	0.353	1.482	2.118 × 10^4^
			**3 N**	337.920	0.784	3.847	3.167 × 10^4^	276.480	0.987	2.978	1.037 × 10^5^
			**5 N**	466.560	0.721	3.990	5.208 × 10^4^	397.440	1.650	4.542	3.516 × 10^5^
			**7 N**	491.520	0.729	5.002	7.951 × 10^4^	426.240	2.483	6.838	6.812 × 10^5^
			**9 N**	562.560	1.138	9.139	1.079 × 10^5^	472.320	2.888	7.927	1.136 × 10^6^
	**Speed**	**7 N**	**120 rpm**	349.440	0.342	2.303	1.165 × 10^4^	301.440	1.119	4.390	1.623 × 10^5^
			**310 rpm**	491.520	0.729	5.002	6.051 × 10^4^	426.240	2.483	6.838	6.812 × 10^5^
			**500 rpm**	602.880	2.405	11.742	4.630 × 10^5^	509.760	2.171	7.579	1.150 × 10^6^
			**690 rpm**	679.680	3.110	18.278	9.708 × 10^5^	569.200	3.444	10.160	2.162 × 10^6^
			**880 rpm**	791.040	4.781	21.920	1.985 × 10^6^	600.960	3.456	11.629	3.416 × 10^6^

## Data Availability

The original contributions presented in this study are included in the article/Supplementary Materials. Further inquiries can be directed to the corresponding authors.

## Supplementary Materials

The following supporting information can be downloaded at: https://www.mdpi.com/article/10.3390/ma18194457/s1. Figure S1: (a) LFP powder sample microstructure, (b) NCM powder samples microstructure; Figure S2: Rheological curve of LFP/NCM slurry; Figure S3: Comparison of wear volume and δ of SUS304 (a, c) ball and (b, d) disk after friction test in LFP slurry system; Figure S4: Comparison of wear volume and δ of SUS304 (a, c) ball and (b, d) disk after friction test in LFP slurry system; Figure S5: Initial surfaces of (a–c) SUS304 balls and (d–f) discs: (a, d) micromorphology, (b, e) EDS analysis of initial blank ball-disc surfaces in LFP slurry system, (c, f) EDS analysis of initial blank ball-disc surfaces in NCM slurry system; Figure S6: SEM Micromorphology and EDS Elemental Composition Analysis of Wear Areas on SUS304 (a–f) Balls and (g–l) Discs in the SUS304-LFP Slurry System Under Different Loads (1, 5, and 9 N) and Rotational Speeds (120, 310, 880 rpm; Figure S7: SEM Micromorphology and EDS Elemental Composition Analysis of Wear Areas on SUS304 (a–f) Balls and (g–l) Discs in the SUS304-NCM Slurry System Under Different Loads (1, 5, and 9 N) and Rotational Speeds (120, 310, 880 rpm); Table S1: EDS Elemental Composition Analysis of Wear Areas on SUS304 Balls and Discs in the SUS304-LFP Slurry System Under Different Operating Conditions; Table S2: EDS Elemental Composition Analysis of Wear Areas on SUS304 Balls and Discs in the SUS304-NCM Slurry System Under Different Operating Conditions.
