# Supplementary material for: Study on the Wear Behavior Mechanism of SUS304 Stainless Steel During the Homogenization Process of LFP/NCM Slurry
Source: Materials (Basel). 2025 Sep 24;18(19):4457. doi: 10.3390/ma18194457 (PMC12525344; doi:10.3390/ma18194457)
Supplement: Supplementary file 1 [file materials-18-04457-s001.zip › materials-3854660-supplementary.pdf]

Study on the Wear Behavior Mechanism of SUS304 Stainless Steel during the Homogenization Process of LFP/NCM Slurry

Xiangli Wen*, Mingkun Bi, Lvzhou Li*, Jianning Ding

Institute of Technology for Carbon Neutralization, Yangzhou University, Yangzhou, 225127, Jiangsu, China.

School of Mechanical Engineering, Yangzhou University, Yangzhou 225127, Jiangsu, China.

*Corresponding author. E-mail: xlwen@yzu.edu.cn, oasis@yzu.edu.cn.

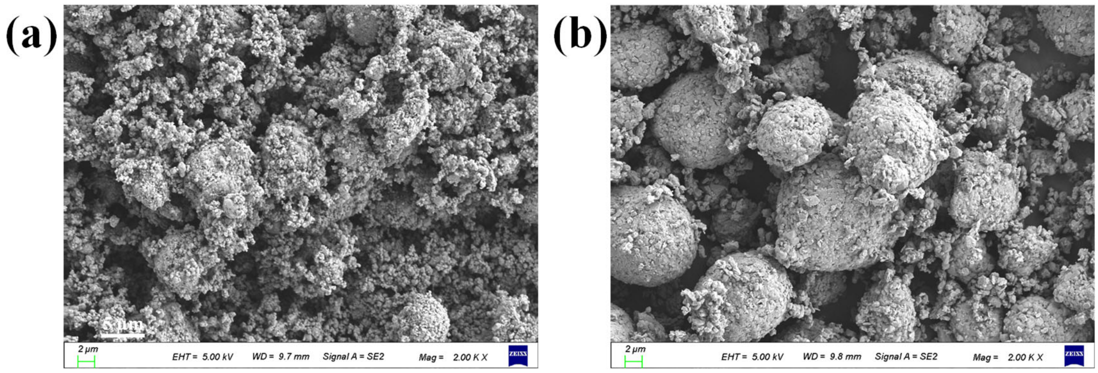

Fig. S1 (a) LFP powder sample microstructure, (b) NCM powder samples microstructure

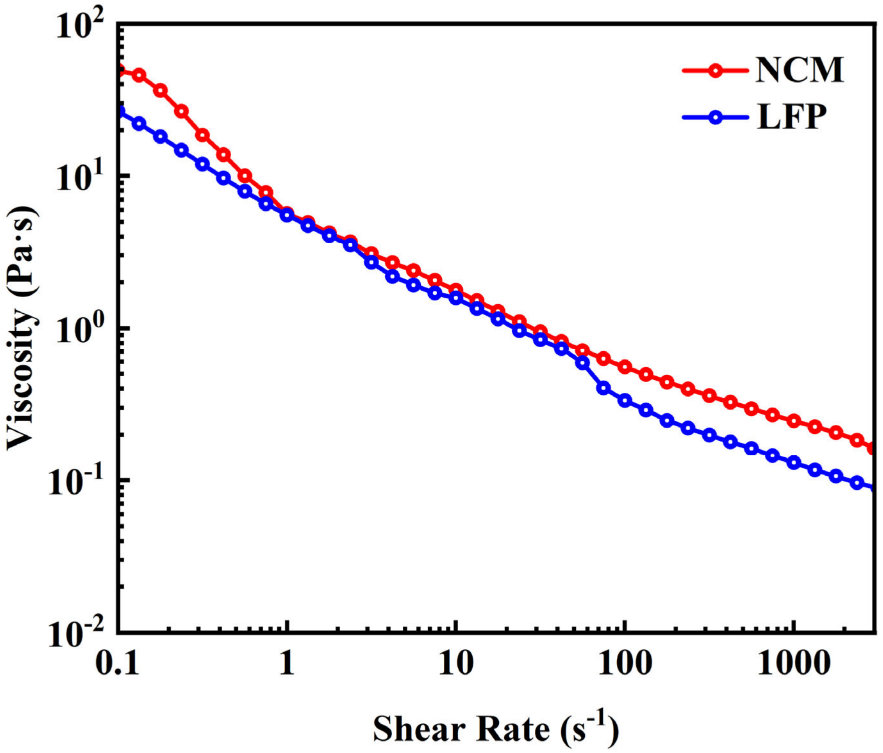

Fig. S2 Rheological curve of LFP/NCM slurry

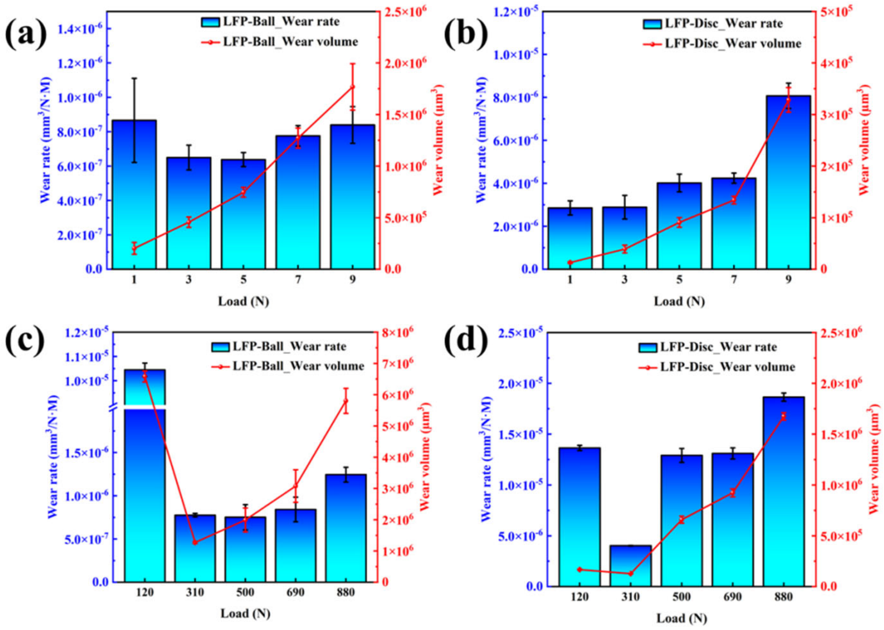

Fig. S3 Comparison of wear volume and δ of SUS304 (a, c) ball and (b, d) disk after friction test in LFP slurry system.

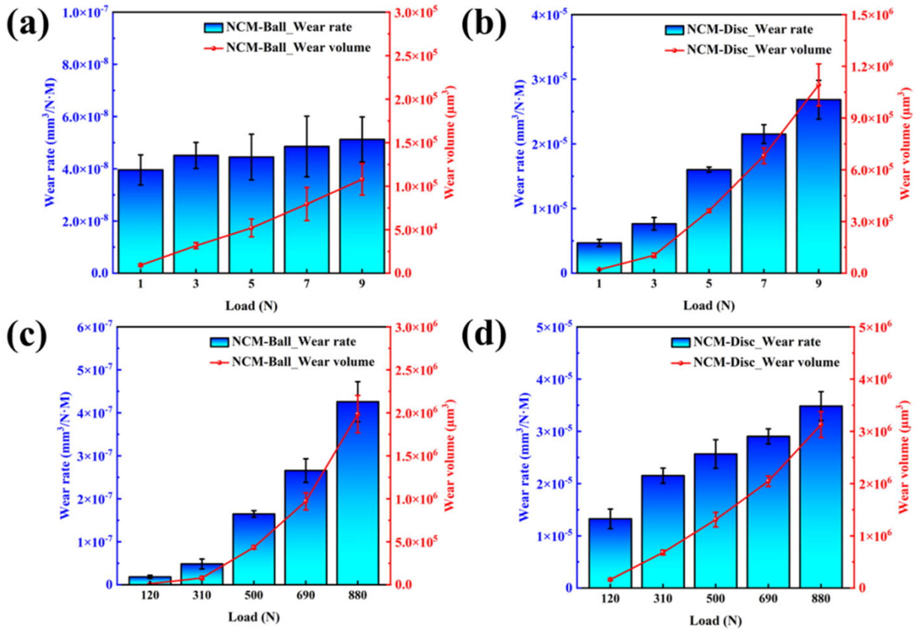

Fig. S4 Comparison of wear volume and δ of SUS304 (a, c) ball and (b, d) disk after friction test in LFP slurry system.

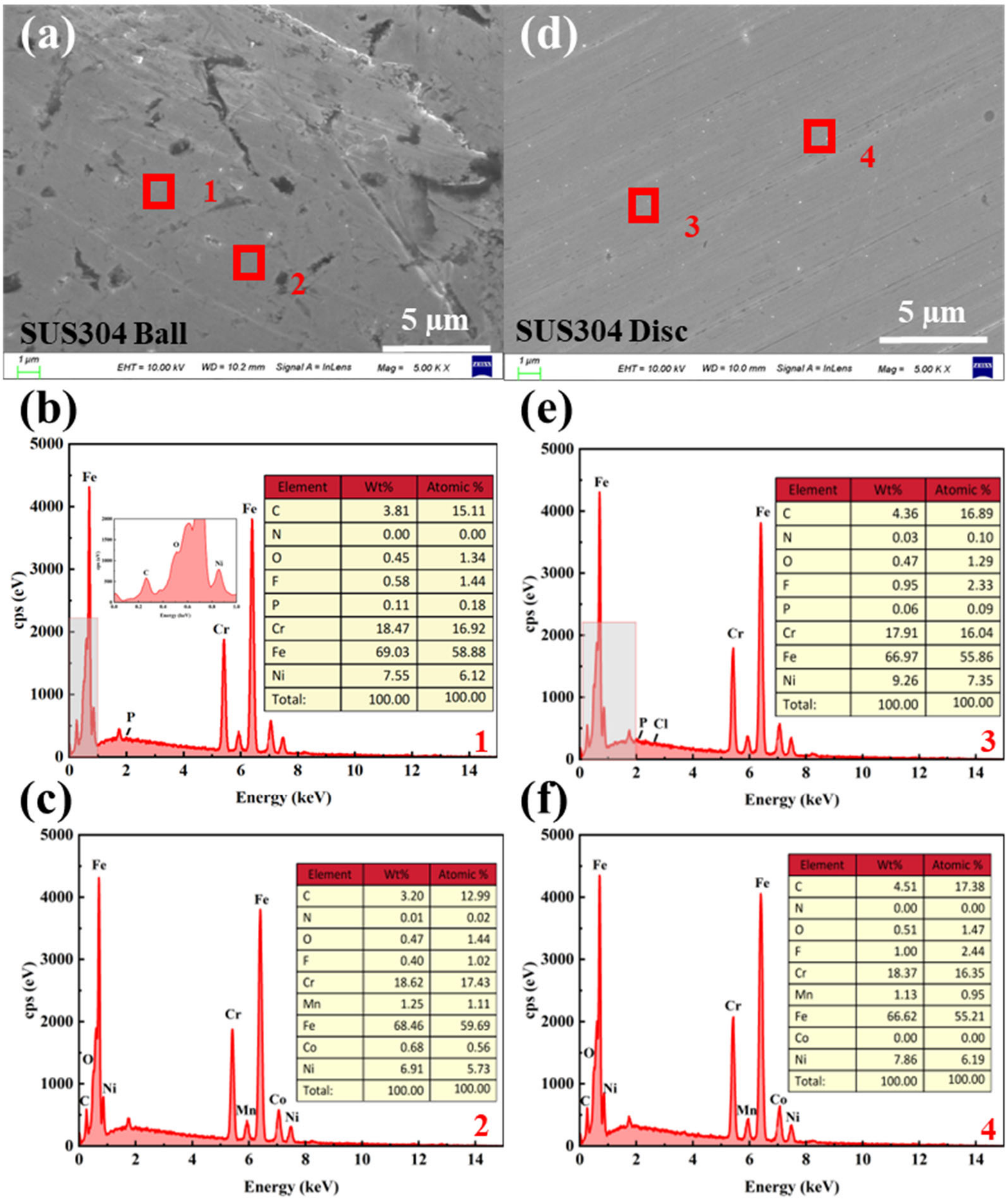

Fig. S5 Initial surfaces of (a-c) SUS304 balls and (d-f) discs: (a, d) micromorphology, (b, e) EDS analysis of initial blank ball-disc surfaces in LFP slurry system, (c, f) EDS analysis of initial blank ball-disc surfaces in NCM slurry system

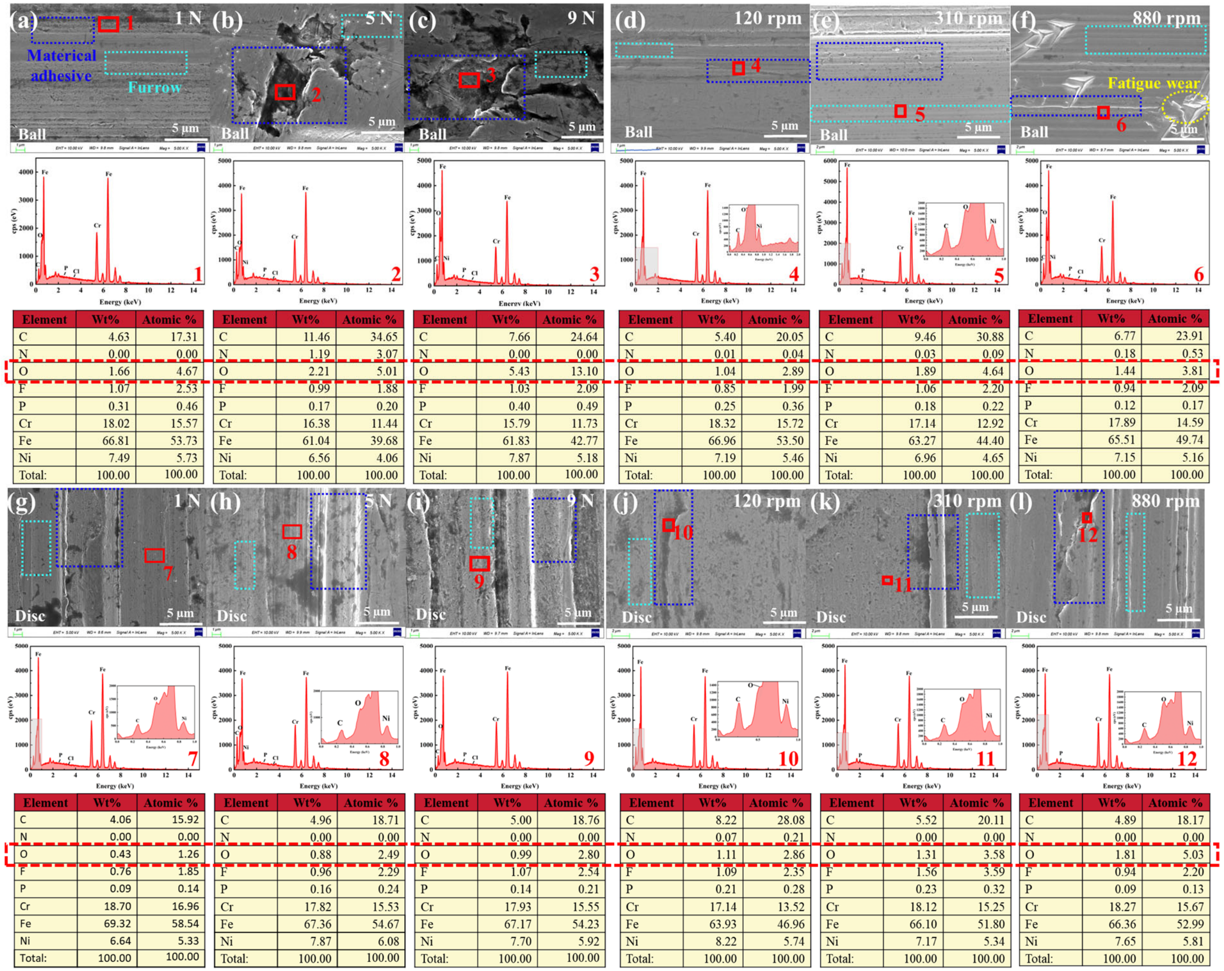

Element	Wt%	Atomic %
C	4.63	17.31
N	0.00	0.00
O	1.66	4.67
F	1.07	2.53
P	0.31	0.46
Cr	18.02	15.57
Fe	66.81	53.73
Ni	7.49	5.73
Total:	100.00	100.00

Element	Wt%	Atomic %
C	11.46	34.65
N	1.19	3.07
O	2.21	5.01
F	0.99	1.88
P	0.17	0.20
Cr	16.38	11.44
Fe	61.04	39.68
Ni	6.56	4.06
Total:	100.00	100.00

Element	Wt%	Atomic %
C	7.66	24.64
N	0.00	0.00
O	5.43	13.10
F	1.03	2.09
P	0.40	0.49
Cr	15.79	11.73
Fe	61.83	42.77
Ni	7.87	5.18
Total:	100.00	100.00

Element	Wt%	Atomic %
C	5.40	20.05
N	0.01	0.04
O	1.04	2.89
F	0.85	1.99
P	0.25	0.36
Cr	18.32	15.72
Fe	66.96	53.50
Ni	7.19	5.46
Total:	100.00	100.00

Element	Wt%	Atomic %
C	9.46	30.88
N	0.03	0.09
O	1.89	4.64
F	1.06	2.20
P	0.18	0.22
Cr	17.14	12.92
Fe	63.27	44.40
Ni	6.96	4.65
Total:	100.00	100.00

Element	Wt%	Atomic %
C	6.77	23.91
N	0.18	0.53
O	1.44	3.81
F	0.94	2.09
P	0.12	0.17
Cr	17.89	14.59
Fe	65.51	49.74
Ni	7.15	5.16
Total:	100.00	100.00

Element	Wt%	Atomic %
C	4.06	15.92
N	0.00	0.00
O	0.43	1.26
F	0.76	1.85
P	0.09	0.14
Cr	18.70	16.96
Fe	69.32	58.54
Ni	6.64	5.33
Total:	100.00	100.00

Element	Wt%	Atomic %
C	4.96	18.71
N	0.00	0.00
O	0.88	2.49
F	0.96	2.29
P	0.16	0.24
Cr	17.82	15.53
Fe	67.36	54.67
Ni	7.87	6.08
Total:	100.00	100.00

Element	Wt%	Atomic %
C	5.00	18.76
N	0.00	0.00
O	0.99	2.80
F	1.07	2.54
P	0.14	0.21
Cr	17.93	15.55
Fe	67.17	54.23
Ni	7.70	5.92
Total:	100.00	100.00

Element	Wt%	Atomic %
C	8.22	28.08
N	0.07	0.21
O	1.11	2.86
F	1.09	2.35
P	0.21	0.28
Cr	17.14	13.52
Fe	63.93	46.96
Ni	8.22	5.74
Total:	100.00	100.00

Element	Wt%	Atomic %
C	5.52	20.11
N	0.00	0.00
O	1.31	3.58
F	1.56	3.59
P	0.23	0.32
Cr	18.12	15.25
Fe	66.10	51.80
Ni	7.17	5.34
Total:	100.00	100.00

Element	Wt%	Atomic %
C	4.89	18.17
N	0.00	0.00
O	1.81	5.03
F	0.94	2.20
P	0.09	0.13
Cr	18.27	15.67
Fe	66.36	52.99
Ni	7.65	5.81
Total:	100.00	100.00

Fig. S6 SEM Micromorphology and EDS Elemental Composition Analysis of Wear Areas on SUS304 (a-f) Balls and (g-l) Discs in the SUS304-LFP Slurry System Under Different Loads (1, 5, and 9 N) and Rotational Speeds (120, 310, 880 rpm)

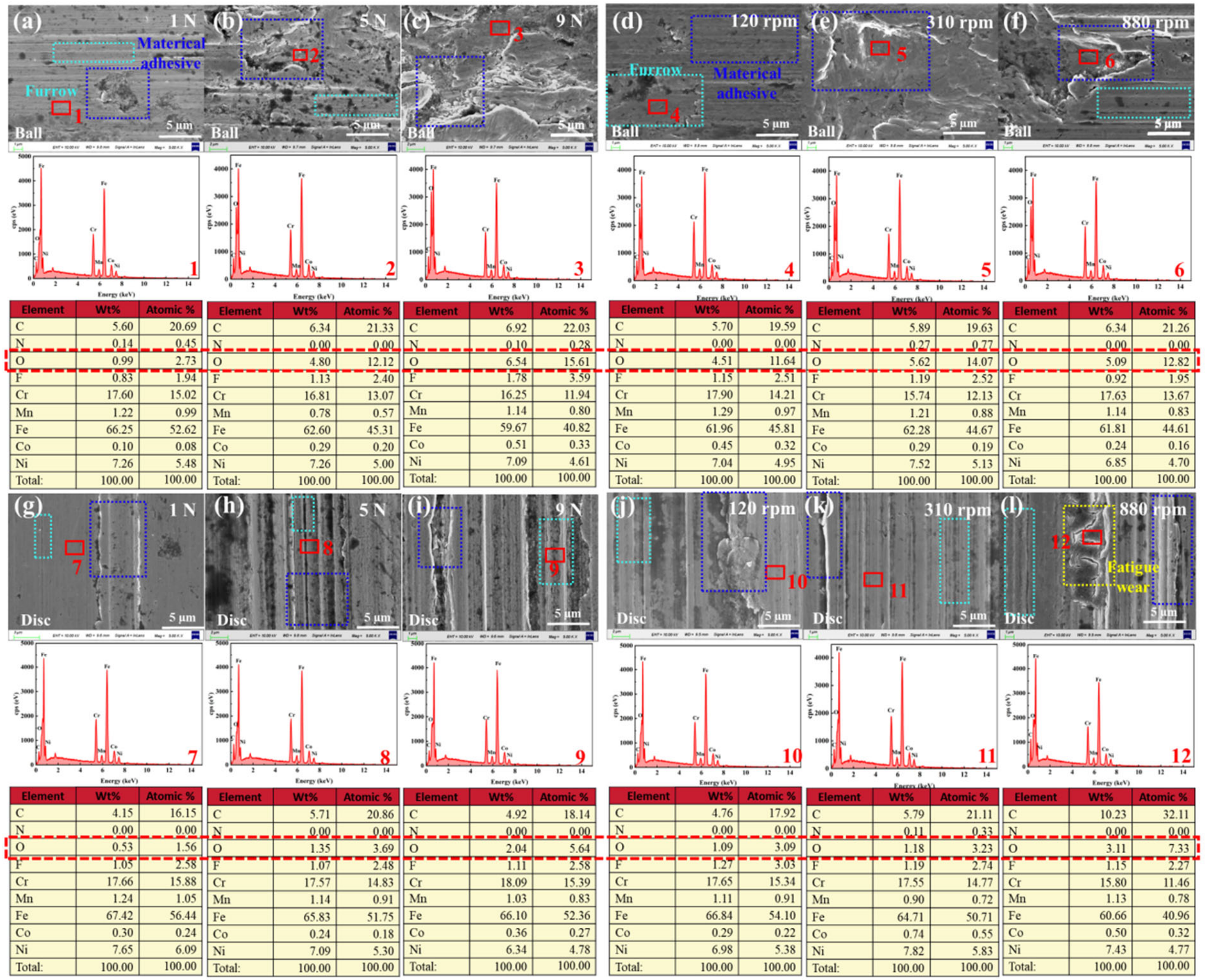

Element	Wt%	Atomic %
C	5.60	20.69
N	0.14	0.45
O	0.99	2.73
F	0.83	1.94
Cr	17.60	15.02
Mn	1.22	0.99
Fe	66.25	52.62
Co	0.10	0.08
Ni	7.26	5.48
Total:	100.00	100.00

Element	Wt%	Atomic %
C	6.34	21.33
N	0.00	0.00
O	4.80	12.12
F	1.13	2.40
Cr	16.81	13.07
Mn	0.78	0.57
Fe	62.60	45.31
Co	0.29	0.20
Ni	7.26	5.00
Total:	100.00	100.00

Element	Wt%	Atomic %
C	6.92	22.03
N	0.10	0.28
O	6.54	15.61
F	1.78	3.59
Cr	16.25	11.94
Mn	1.14	0.80
Fe	59.67	40.82
Co	0.51	0.33
Ni	7.09	4.61
Total:	100.00	100.00

Element	Wt%	Atomic %
C	5.70	19.59
N	0.00	0.00
O	4.51	11.64
F	1.15	2.51
Cr	17.90	14.21
Mn	1.29	0.97
Fe	61.96	45.81
Co	0.45	0.32
Ni	7.04	4.95
Total:	100.00	100.00

Element	Wt%	Atomic %
C	5.89	19.63
N	0.27	0.77
O	5.62	14.07
F	1.19	2.52
Cr	15.74	12.13
Mn	1.21	0.88
Fe	62.28	44.67
Co	0.29	0.19
Ni	7.52	5.13
Total:	100.00	100.00

Element	Wt%	Atomic %
C	6.34	21.26
N	0.00	0.00
O	5.09	12.82
F	0.92	1.95
Cr	17.63	13.67
Mn	1.14	0.83
Fe	61.81	44.61
Co	0.24	0.16
Ni	6.85	4.70
Total:	100.00	100.00

Element	Wt%	Atomic %
C	4.15	16.15
N	0.00	0.00
O	0.53	1.56
F	1.05	2.58
Cr	17.66	15.88
Mn	1.24	1.05
Fe	67.42	56.44
Co	0.30	0.24
Ni	7.65	6.09
Total:	100.00	100.00

Element	Wt%	Atomic %
C	5.71	20.86
N	0.00	0.00
O	1.35	3.69
F	1.07	2.48
Cr	17.57	14.83
Mn	1.14	0.91
Fe	65.83	51.75
Co	0.24	0.18
Ni	7.09	5.30
Total:	100.00	100.00

Element	Wt%	Atomic %
C	4.92	18.14
N	0.00	0.00
O	2.04	5.64
F	1.11	2.58
Cr	18.09	15.39
Mn	1.03	0.83
Fe	66.10	52.36
Co	0.36	0.27
Ni	6.34	4.78
Total:	100.00	100.00

Element	Wt%	Atomic %
C	4.76	17.92
N	0.00	0.00
O	1.09	3.09
F	1.27	3.03
Cr	17.65	15.34
Mn	1.11	0.91
Fe	66.84	54.10
Co	0.29	0.22
Ni	6.98	5.38
Total:	100.00	100.00

Element	Wt%	Atomic %
C	5.79	21.11
N	0.11	0.33
O	1.18	3.23
F	1.19	2.74
Cr	17.55	14.77
Mn	0.90	0.72
Fe	64.71	50.71
Co	0.74	0.55
Ni	7.82	5.83
Total:	100.00	100.00

Element	Wt%	Atomic %
C	10.23	32.11
N	0.00	0.00
O	3.11	7.33
F	1.15	2.27
Cr	15.80	11.46
Mn	1.13	0.78
Fe	60.66	40.96
Co	0.50	0.32
Ni	7.43	4.77
Total:	100.00	100.00

Fig. S7 SEM Micromorphology and EDS Elemental Composition Analysis of Wear Areas on SUS304 (a-f) Balls and (g-l) Discs in the SUS304-NCM Slurry System Under Different Loads (1, 5, and 9 N) and Rotational Speeds (120, 310, 880 rpm)

Table S1 EDS Elemental Composition Analysis of Wear Areas on SUS304 Balls and Discs in the SUS304-LFP Slurry System Under Different Operating Conditions

**Type**	**Element**	**Weight percentage (wt.%)**						
		**Initial**	**1 N**	**5 N**	**9 N**	**120 rpm**	**310 rpm**	**880 rpm**
**Ball**	**C**	3.81	4.63	11.46	7.66	5.40	9.46	8.23
	**N**	0.00	0.00	1.19	0.00	0.01	0.03	0.03
	**O**	**0.45**	**1.66**	**2.21**	**5.43**	**1.04**	**1.89**	**0.79**
	**F**	0.58	1.07	0.99	1.03	0.85	1.06	0.82
	**Cr**	0.11	0.31	0.17	0.40	0.25	0.18	0.04
	**Mn**	18.47	18.02	16.38	15.79	18.32	17.14	17.61
	**Fe**	69.03	66.81	61.04	61.83	66.96	63.27	64.82
	**Co**	7.55	7.49	6.56	7.87	7.19	6.96	7.67
**Disc**	**C**	4.36	4.06	4.96	5.00	8.22	5.52	4.89
	**N**	0.03	0.00	0.00	0.00	0.07	0.00	0.00
	**O**	**0.47**	**0.43**	**0.88**	**0.99**	**1.11**	**1.31**	**1.81**
	**F**	0.95	0.76	0.96	1.07	1.09	1.56	0.94
	**Cr**	0.06	0.09	0.16	0.14	0.21	0.23	0.09
	**Mn**	17.91	18.70	17.82	17.93	17.14	18.12	18.27
	**Fe**	66.97	69.32	67.36	67.17	63.93	66.10	66.36
	**Co**	9.26	6.64	7.87	7.70	8.22	7.17	7.65

Table S2 EDS Elemental Composition Analysis of Wear Areas on SUS304 Balls and Discs in the SUS304-NCM Slurry System Under Different Operating Conditions

**Type**	**Element**	**Weight percentage (wt.%)**						
		**Initial**	**1 N**	**5 N**	**9 N**	**120 rpm**	**310 rpm**	**880 rpm**
**Ball**	**C**	3.20	5.60	6.34	6.92	5.70	5.89	6.34
	**N**	0.01	0.14	0.00	0.10	0.00	0.27	0.00
	**O**	**0.47**	**0.99**	**4.80**	**6.54**	**4.51**	**5.62**	**5.09**
	**F**	0.40	0.83	1.13	1.78	1.15	1.19	0.92
	**Cr**	18.62	17.60	16.81	16.25	17.90	15.74	17.63
	**Mn**	1.25	1.22	0.78	1.14	1.29	1.21	1.14
	**Fe**	68.46	66.25	62.60	59.67	61.96	62.28	61.81
	**Co**	0.68	0.10	0.29	0.51	0.45	0.29	0.24
	**Ni**	6.91	7.26	7.26	7.09	7.04	7.52	6.85
**Disc**	**C**	4.51	4.15	5.71	4.92	4.76	5.79	10.23
	**N**	0.00	0.00	0.00	0.00	0.00	0.11	0.00
	**O**	**0.51**	**0.53**	**1.35**	**2.04**	**1.09**	**1.18**	**3.11**
	**F**	1.00	1.05	1.07	1.11	1.27	1.19	1.15
	**Cr**	18.37	17.66	17.57	18.09	17.65	17.55	15.80
	**Mn**	1.13	1.24	1.14	1.03	1.11	0.90	1.13
	**Fe**	66.62	67.42	65.83	66.10	66.84	64.71	60.66
	**Co**	0.00	0.30	0.24	0.36	0.29	0.74	0.50
	**Ni**	7.86	7.65	7.09	6.34	6.98	7.82	7.43
